# Can We Make Spine Surgery Safer and Better?

**DOI:** 10.3390/jcm11123400

**Published:** 2022-06-13

**Authors:** Rafael De la Garza Ramos

**Affiliations:** Department of Neurological Surgery, Montefiore Medical Center, Albert Einstein College of Medicine, New York, NY 10467, USA; rdelag@montefiore.org

Driven mostly by an aging population, the utilization of spine surgery has increased exponentially over the last decades [1]. Chronic low back pain and spinal deformity continue to be leading causes of disability [2,3], and cancer patients with spinal metastases are living longer due to advancements in systemic and targeted therapies [4]. However, the management of complex spinal disorders continues to be challenging. Although the benefits of surgical intervention for deformity and spinal tumors have been well documented, these procedures are costly and associated with significant morbidity [5,6,7,8]. As such, finding value by improving safety and outcomes in treatment of these conditions remains a priority within our field and will most likely derive from multidisciplinary approaches, advanced techniques/enabling technologies, and predictive analytics.

Patient-centered care and the use of multidisciplinary collaborations inside and outside of the operating room is a cornerstone of safe and effective management of spinal disorders. Multiple studies have shown the benefits of multidisciplinary case conferences, tumor boards, and shared-decision making in the management of these conditions [9,10]. Naidu et al. recently showed that a multidisciplinary spine clinic significantly reduced the utilization of surgery as a first treatment option and provided quicker times to injections for patients afflicted with chronic back and/or leg pain [11]. A systematic review of ten studies examining multidisciplinary approaches in spinal deformity found that these strategies significantly reduced operative time and complication occurrence [9]. Similarly, Nguyen et al. showed that a multidisciplinary team approach consisting of neurosurgery, orthopedic oncology, plastic surgery, medical oncology, and radiation oncology was able to optimize surgical outcomes, reduce morbidity, and improve care and satisfaction in high-risk patients undergoing oncological spine surgery [12].

The second strategy to improve safety and outcomes includes the use of advanced techniques and enabling technologies. Anand et al. reported a 13-year experience treating severe adult spinal deformity with minimally invasive circumferential strategies without posterior osteotomies [13]. They examined 136 patients with major deformity and found a significant improvement in quality of life with a low rate of proximal junctional failure (2.2%) and pseudarthrosis (5.9%) [13]. In patients suffering from spinal tumors, minimally invasive techniques such as percutaneous fixation, hybrid/tubular approaches, and others have resulted in reduced blood loss, reduced perioperative morbidity, and favorable outcomes as well [14,15]. Echt et al. compared the minimally invasive approach to the traditional approach in patients undergoing separation surgery for metastatic disease, finding that the former was associated with a significant reduction in blood loss and a comparable neurological outcome [15]. Other exciting technologies that have enormous potential include robotics, augmented reality, and virtual reality. 

The third aspect that will most likely improve the safety and outcome of complex spine surgery is the use of advanced predictive analytics, particularly artificial intelligence. Machine and deep-learning algorithms can offer high predictive capability and can aid in complication prediction, survival prediction, and the identification of high-risk patients [16,17,18,19]. Patients that are predicted to have a poor outcome or a major complication could receive a modified treatment plan or be optimized preoperatively.

The field of spinal surgery is rapidly changing and we must adapt to it. As specialists dealing with such complex conditions, we must focus on providing humanized and personalized high-quality care to our patients. The use of the approaches described herein will most certainly help us achieve that. 
